# Supplementary material for: Inhibition of Succinate Dehydrogenase by Pesticides (SDHIs) and Energy Metabolism
Source: Int J Mol Sci. 2023 Feb 17;24(4):4045. doi: 10.3390/ijms24044045 (PMC9962667; doi:10.3390/ijms24044045)
Supplement: Supplementary file 1 [file ijms-24-04045-s001.zip › ijms-2155639-supplementary.pdf]

Supplemental S1

Succinate accumulation, a quantitative model.

The consequences of the engagement of this salvage pathway could be examined with a simple model. To highlight the possible relevance of this pathway we shall examine the situation of fatty acid oxidation: The beta oxidation step releases one NADH and one $FADH_2$ to generate acetyl Co-A, which is oxidized to CO_2 by the TCA cycle. Then in presence of the SDH activity the oxidation scheme for each two carbons from a saturated amino acid will be as follow (Figure S1).

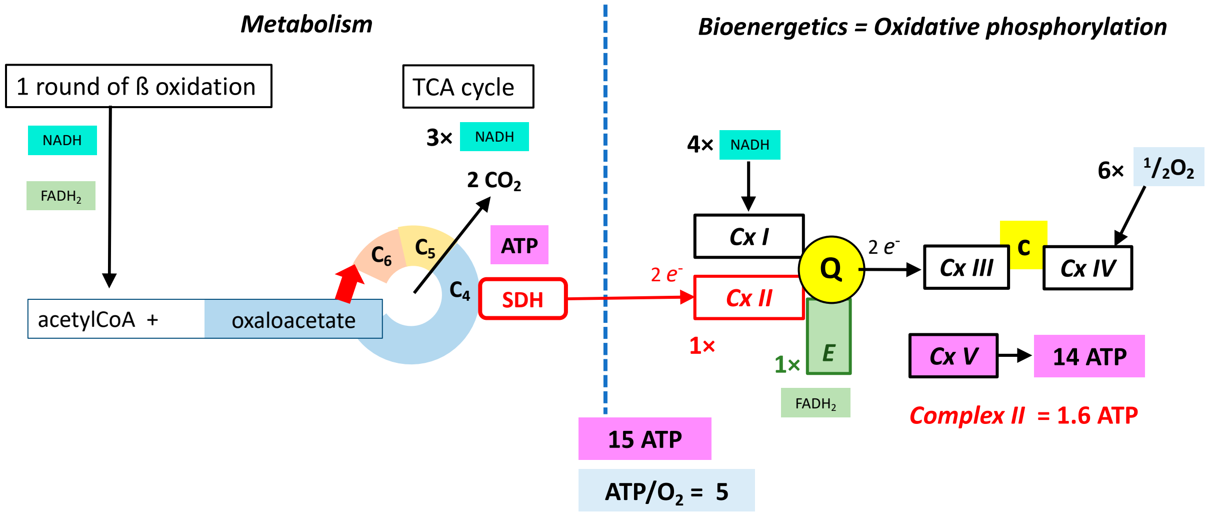

Figure S1

"*E*" is the electron transfer flavoprotein (ETF) that reoxidizes FADH2 from beta-oxidation

If SDH activity is lost and compensated by regeneration of oxaloacetate from the pyruvate derived from glucose (reaction 3 in Figure 10-3), the biochemical scheme becomes as shown in Figure S2. From the point of view of oxidative phosphorylation, the result is a moderately decreased (-11%) ATP yield for each round (acetyl-CoA); but at the opposite, the yield in ATP per oxygen is increased by 7%.

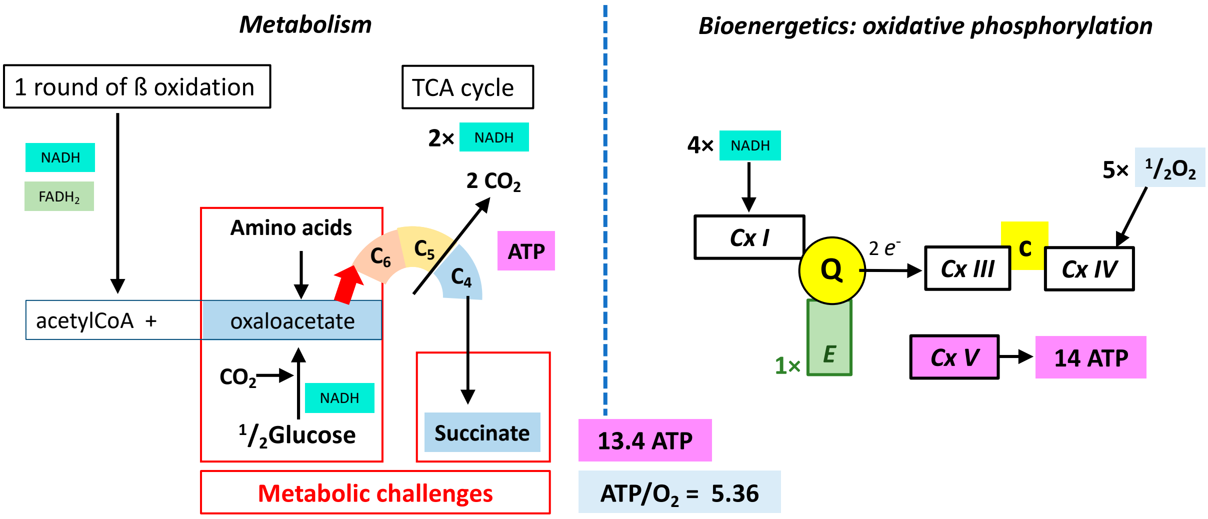

Figure S2

The metabolic challenges are oxaloacetate supply and succinate release/accumulation

A simple model would allow to imagine the consequences of the recruitment of this salvage pathway when cells are subject to a gradual decrease in the flux of the SDH reaction (Figure 10-6). The efficiency of this salvage pathway as inhibition increases is dependent on several determinant factors listed in the figure.

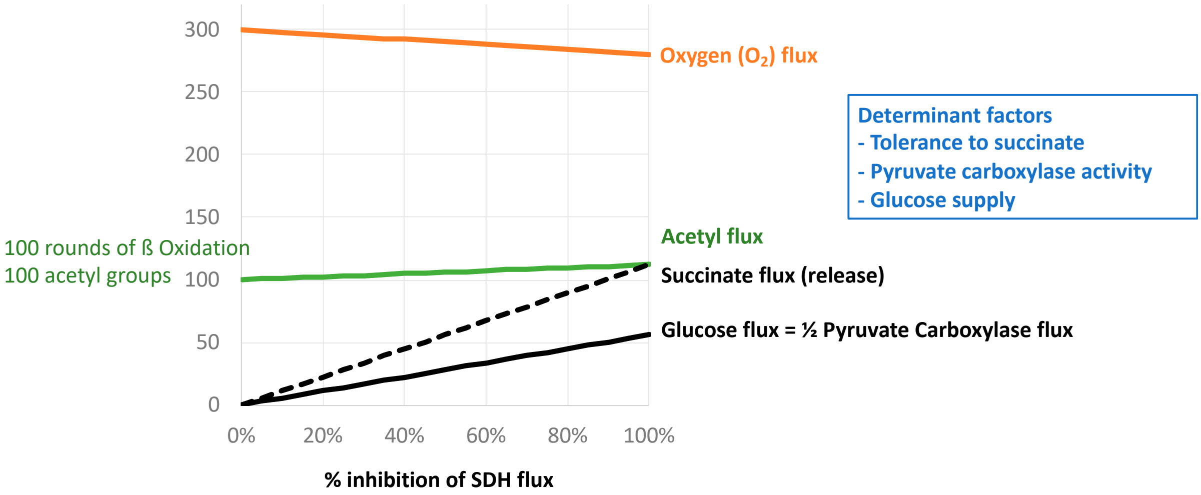

Figure S3

The model considers as a starting point (0% inhibition of SDH) the ATP production resulting from 100 rounds of beta oxidation with oxidation in the TCA of the 100 acetyl Co-A. As SDH inhibition progresses the salvage pathway (figure 10-5) is recruited to maintain a constant ATP production rate. Oxygen consumption and fatty acid oxidation are marginally modified. In contrast, the recruitment of glucose and generation of succinate increase from zero to values representing 50% and 100% respectively of the fatty acid oxidation rate.

Supplemental S2

Kinetic compensation and expression level of SDH.

In the subsequent model we suppose that if under normal condition SDH oxidation flux implies X% of maximal SDH activity present the inhibition of 100%-X% is required before other mechanism than kinetic inhibition is required. This generates a threshold effect, with abrupt transition from no consequences for the relative SDH inhibition to a situation in which the consequences shown in Figure S4 develop linearly; and this within a range shifted to higher values and narrower range of inhibition as excess in SDH activity increases. The values for excess in SDH (150-500%) explore a realistic range as the total energy expenditure (TEE) of a person over 24 hours ranges between 1.4 and 2.4 times the basal metabolic rate (BMR); and moreover, similar complex II and complex I enzymatic activities compared to metabolic oxidation pathways suggests up to five-times higher maximal SDH activity than required to match with contribution of complex I. For clarity, only the use of glucose is considered; in absence of SDH a basal rate of glucose is supposed for replenishment in oxaloacetate. This could be considered as a realistic starting point, and allows to evaluate, in relative terms, how the recruitment of the salvage pathway would impact on glucose consumption.

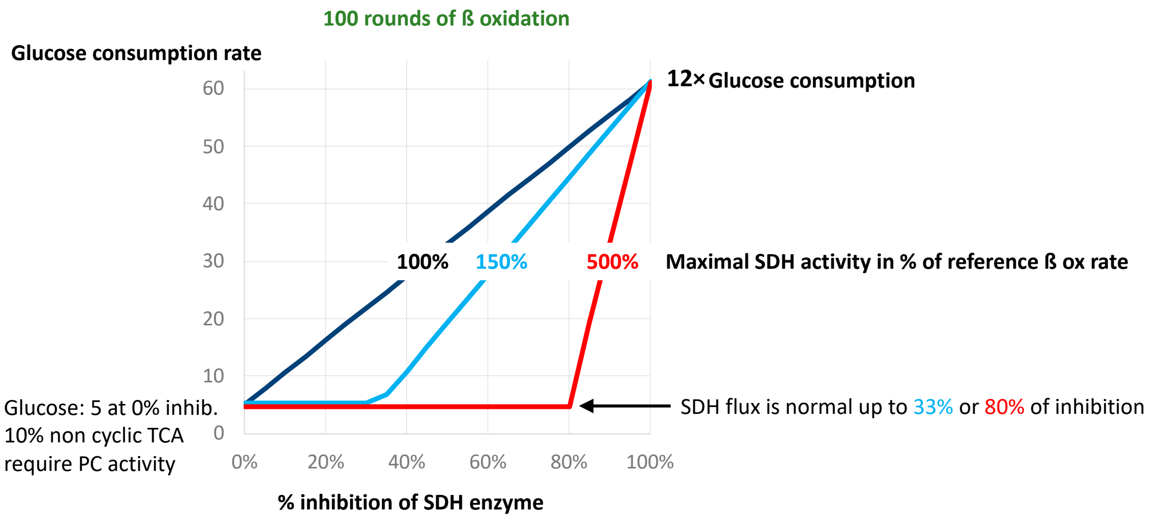

Figure S4

The model is the same as above except that 1) a low flux of glucose is supposed to be present in absence of inhibition. 2) the maximal SDH activity is considered with three different levels relative to the physiological needs: 100% no excess in SDH (as above); 150%, excess by 50%; 500%, five times excess in SDH activity. Then for a given relative inhibition of SDH activity (X-axis) if the remaining SDH activity remains above the physiological need (100%), there is no inhibition of the SDH flux; If not, the rules of figure 10-6 apply.

This oversimplified model makes apparent:

- Why/how **a threshold of inhibition is required to observe consequences**.
- That within a range **this threshold effect enhances the contrast between individuals** if they show different expression levels for SDH, the range of inhibition 33-80% impacts on the blue trace (150% SDH) and not on the red (500% SDH).
- Why **stimulation** that lowers the “reserve of SDH activity” **would aggravate the impact of a same inhibition level**.

- That the **salvage pathway uses more of the normal resources (glucose amino acids) and generates a new waste (succinate)**.
- That an **appropriate response** to SDH inhibitor **would be an increase in SDH expression**.
- If one considers that 500% represents **the maximal possible increase** in SDH activity it **would define a maximal limit to the adaptive response** hence a range in which allostasis (preconditioning) remains possible. Then if the 100%, 150% and 500% SDH expression levels reflect the heterogeneity of a natural population, the adaptive response to the inhibitor would restrict this variability.

Supplemental S3

Lactate and succinate reoxidation

Plasmatic levels of succinate are low (µM) and variable hence two to three orders of magnitude lower than that of lactate (mM) a marginal activity of the succinate cycle or/and the proximal oxidation of succinate may explain this. The comparison of lactate or succinate reoxidation schemes may explain this.

Lactate oxidation pathway reproduces that of glucose/pyruvate although absence of glycolytic ATP formation steps and reoxidation of one more NADH than pyruvate lowers ATP/O_2 (reimbursement of the oxygen debt). In contrast succinate accumulated during hypoxia is intensely reoxidized by SDH and the intense complex II activity reduces strongly quinone and restrains NADH reoxidation by complex I (hence all other metabolic oxidation pathways) and even more forces reverse electron transfer in complex I (oxidation of quinone with reduction of NAD).

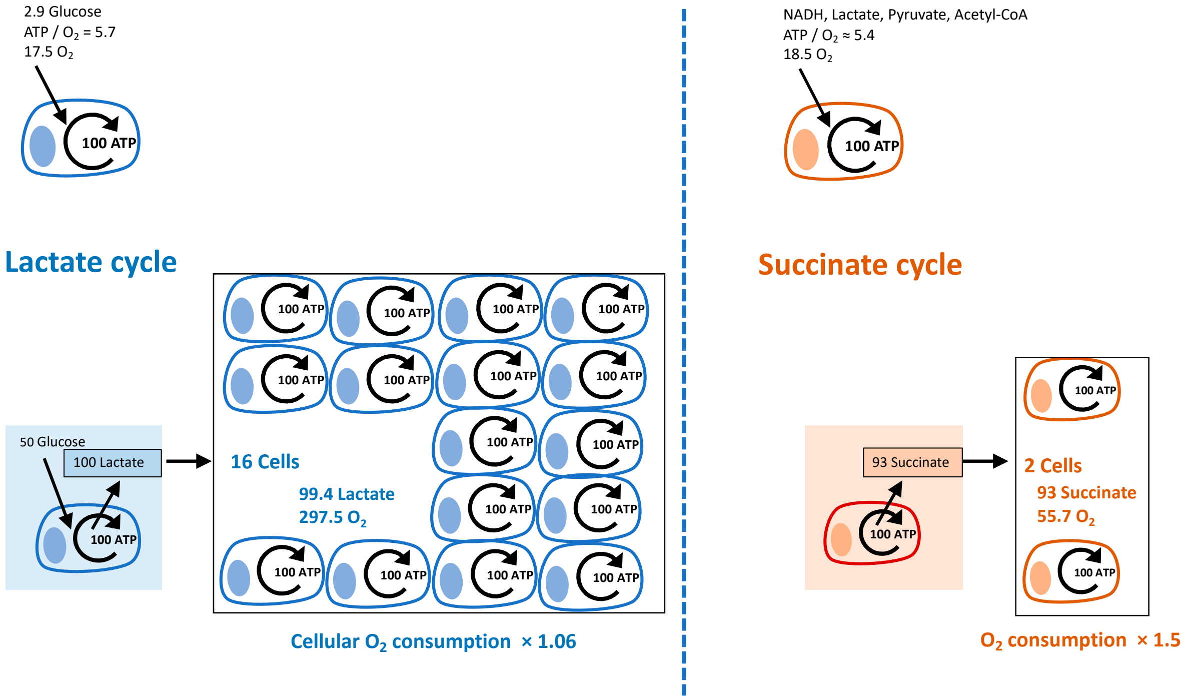

Figure S5

Aerobic / anaerobic lactate or succinate cycles:

Top: reference aerobic metabolism with full oxidation (into CO_2) of glucose (left/blue) and of other substrates (right/orange), this is made with purpose to enlighten that succinate generation could sustain ATP production from other sources than glucose. The theoretical ATP/O_2 values are indicated with the oxygen consumption associated to the generation (turnover) of 100 ATP molecules.

Bottom: shadowed areas figure anaerobic metabolism during one round (100 ATP molecules) with lactic fermentation (blue) or succinate generation (orange). The boxed areas represent the number of rounds/cells implied in the reimbursement of the oxygen debt generated by the release of lactate or succinate during the anaerobic period. The full oxidation of lactate could feed 100% of ATP need for 16 rounds/cells. With succinate the result depends on the scheme adopted for oxidation: the complete oxidation would yield figures slightly different from that of lactate. However, in presence of relatively high concentration of succinate (vicinity of the succinate releasing site) the fierce competition exerted by *complex II* against *complex I* leads to consider also (essentially) a partial oxidation based on the activity of *complex II* exclusively (Succinate to fumarate/malate). Then the poor yield in ATP per succinate results in drastically different figures and the succinate released by the anaerobic period can feed only two oxidative rounds/cells. A direct consequence is that succinate would be oxidized proximal to its generation site and with a significantly increased local oxygen consumption. The end-

product (likely malate) could be considered as equivalent to (and possibly converted into) lactate for subsequent full oxidation. Or reused by the anaerobic cell if a succinate cycle is considered.

Actually, this issue of impact of succinate generation on neighboring cells is dependent on the permeation of succinate across the plasma membrane. This permeability is likely to be low in comparison to possible SDH activity. On one hand this would further restrict the extent of succinate oxidation outside of the production site/cell, on the other hand once released in the external medium succinate would be somehow protected from fast oxidation and travel over longer distances. Restricted permeability of the plasma membrane would make pertinent to consider the situation within a single cell, in which a transiently hypoxic subcellular domain would initiate the conversion of the whole cell to succinate metabolism (intracellular succinate cycle) with higher succinate levels, mitochondrial ROS release, and subsequent signaling cascade.

Supplemental S4

Lack of evaluation of the impact of SDHIs on metabolic diseases presently.

SDHIs have not been extensively used in the academic field. The result is a clear lack of knowledge exemplified by search in Medline (figure S6, search in June 2021).

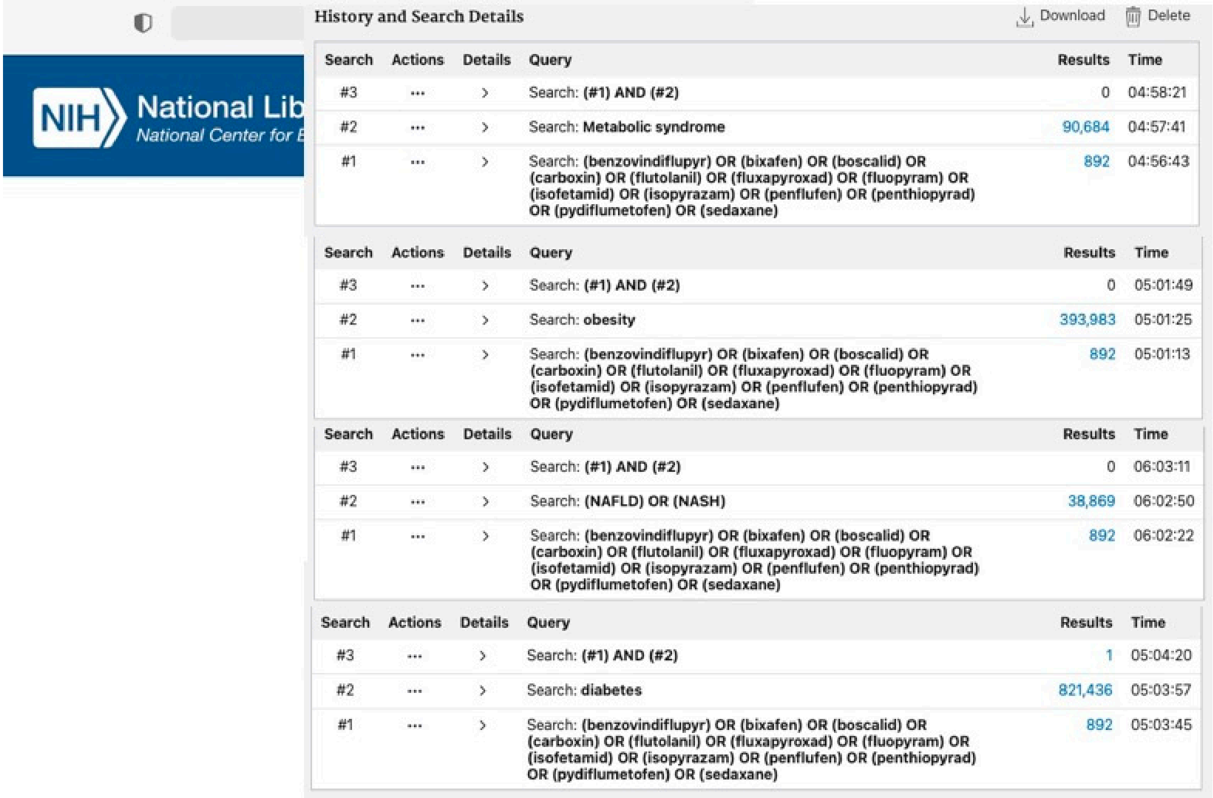

Search	Actions	Details	Query	Results	Time
#3	...	>	Search: (#1) AND (#2)	0	04:58:21
#2	...	>	Search: Metabolic syndrome	90,684	04:57:41
#1	...	>	Search: (benzovindiflupyr) OR (bixafen) OR (boscalid) OR (carboxin) OR (flutolanil) OR (fluxapyroxad) OR (fluopyram) OR (isofetamid) OR (isopyrazam) OR (penflufen) OR (penthiopyrad) OR (pydiflumetofen) OR (sedaxane)	892	04:56:43

Search	Actions	Details	Query	Results	Time
#3	...	>	Search: (#1) AND (#2)	0	05:01:49
#2	...	>	Search: obesity	393,983	05:01:25
#1	...	>	Search: (benzovindiflupyr) OR (bixafen) OR (boscalid) OR (carboxin) OR (flutolanil) OR (fluxapyroxad) OR (fluopyram) OR (isofetamid) OR (isopyrazam) OR (penflufen) OR (penthiopyrad) OR (pydiflumetofen) OR (sedaxane)	892	05:01:13

Search	Actions	Details	Query	Results	Time
#3	...	>	Search: (#1) AND (#2)	0	06:03:11
#2	...	>	Search: (NAFLD) OR (NASH)	38,869	06:02:50
#1	...	>	Search: (benzovindiflupyr) OR (bixafen) OR (boscalid) OR (carboxin) OR (flutolanil) OR (fluxapyroxad) OR (fluopyram) OR (isofetamid) OR (isopyrazam) OR (penflufen) OR (penthiopyrad) OR (pydiflumetofen) OR (sedaxane)	892	06:02:22

Search	Actions	Details	Query	Results	Time
#3	...	>	Search: (#1) AND (#2)	1	05:04:20
#2	...	>	Search: diabetes	821,436	05:03:57
#1	...	>	Search: (benzovindiflupyr) OR (bixafen) OR (boscalid) OR (carboxin) OR (flutolanil) OR (fluxapyroxad) OR (fluopyram) OR (isofetamid) OR (isopyrazam) OR (penflufen) OR (penthiopyrad) OR (pydiflumetofen) OR (sedaxane)	892	05:03:45

Figure S6
